# Quantitation of global histone post-translational modifications reveal anti-inflammatory epigenetic mechanisms of liquiritigenin based on the optimized super-SILAC strategy

**DOI:** 10.3389/fcell.2025.1566567

**Published:** 2025-03-27

**Authors:** Ping Liu, Jun Zhang, Jingdan Zhang, Yucheng Yuan, Zhiqing Liu, Sixian Chen, Kaifeng Chen, Li Dong, Zhiyuan Cheng, Yinan Zhang, Meiyu Geng, Minjia Tan, Wensi Zhao, Dong Xie

**Affiliations:** ^1^ School of Chinese Materia Medica, Nanjing University of Chinese Medicine, Nanjing, Jiangsu, China; ^2^ Shanghai Institute of Materia Medica, Chinese Academy of Sciences, Shanghai, China; ^3^ University of Chinese Academy of Sciences, Beijing, China; ^4^ School of Pharmaceutical Sciences, Southern Medical University, Guangzhou, China; ^5^ Zhongshan Institute for Drug Discovery, Shanghai Institute of Materia Medica, Chinese Academy of Sciences, Zhongshan, China; ^6^ State Key Laboratory of Mathematical Science, Academy of Mathematics and Systems Science, Chinese Academy of Sciences, Beijing, China; ^7^ Department of Thoracic Surgery, Shanghai Pulmonary Hospital, School of Medicine, Tongji University, Shanghai, China

**Keywords:** histone post-translational modifications, super-SILAC, liquiritigenin, inflammation, epigenetics, PPAR

## Abstract

Liquiritigenin (LIQ) is a dihydroflavonone monomer compound with a planar ring structure that exhibits potent anti-inflammatory activity. The post-translational modifications (PTMs) of histones are closely associated with inflammatory diseases. To explore the relationships between the anti-inflammatory effects and epigenetic regulatory mechanisms of LIQ, we optimized the super stable isotope labeling by amino acids in cell culture (super-SILAC) method combined with a compound stimulation strategy. Moreover, we evaluated the identification coverage and demonstrated high reliability as well as reproducibility of the optimized method at both the peptide and cellular lysate levels, which are promising for elucidating disease pathology and drug mechanisms. We further applied the method to a system-wide characterization of histone PTMs in M1 macrophages treated with LIQ. The quantitative results showed that H4K5ac, H4K16ac, H3K9ac, H3K27ac, and H2BK12ac are significantly upregulated. Transcriptome analysis revealed that LIQ could exert anti-inflammatory effects by modulating the histone PTMs and regulating gene expressions through the peroxisome proliferator-activated receptor (PPAR) signaling pathway. Collectively, we provide a sensitive and universal strategy for research on the epigenetic mechanisms of natural products as well as facilitate epigenetic understanding of LIQ in inflammatory therapies.

## Graphical Abstract

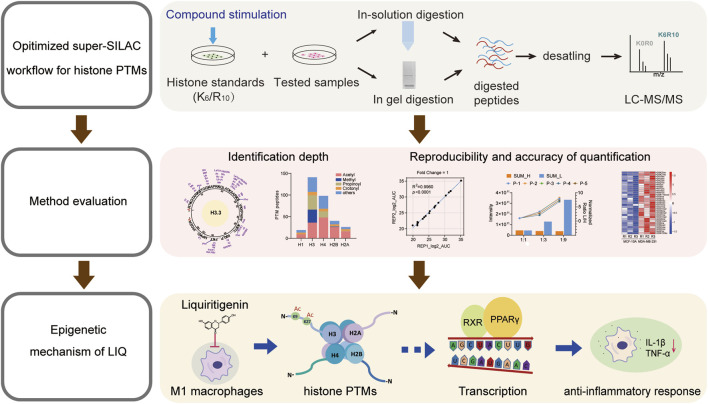

## Introduction

Natural products with structural diversity often exhibit therapeutic activities against various diseases (Baker et al., 2007; Zhao et al., 2023). Approximately 25%–50% of commercially marketed drugs are derived from natural products (Kingston, 2011). Flavonoid compounds play important roles in anti-inflammatory activities through their planar ring structures, which make them promising lead compounds for inflammatory therapy (Theoharides et al., 2001; Wang et al., 2021; Li et al., 2023). Previous studies have revealed that most flavonoids and their derivative dihydroflavones exert anti-inflammatory effects by reducing the expressions of proinflammatory cytokines like IL-6, IL-8, TNF-α, and IL-1β through the NF-κB, MAPK, and JNK-STAT pathways (Serafini et al., 2010; Santangelo et al., 2007). Liquiritigenin (LIQ) is a dihydroflavonone monomer compound isolated from licorice that has been demonstrated to have potent anti-inflammatory effects through inhibition of the activation of NF-κB in macrophages as well as reduction of the production of inducible nitric oxide synthase (iNOS) and proinflammatory cytokines (Babu et al., 2023; Kim et al., 2008; Wang et al., 2015). Additionally, LIQ positively modulates the activity of sirtuin 3 (SIRT3), which is one of the most prominent deacetylases (Zhou et al., 2022). Nonetheless, the relationships between the anti-inflammatory effects of LIQ and regulation of its epigenetic mechanisms remain unclear.

Epigenetic regulation plays a crucial role in the regulation of gene expression. Epigenetic marks can alter the chromatin structure, thereby influencing gene expressions and cellular functions as well as participating in the pathogenesis of various diseases (Zhang and Cao, 2021; Zhu et al., 2021). Histone post-translational modifications (PTMs) are critical epigenetic regulatory factors that are regulated in a highly dynamic and complicated manner owing to the enzymes that catalyze the addition of specific PTMs (writers), reader proteins that recognize and bind specific domains (readers), and enzymes that remove PTMs (erasers) (Millán-Zambrano et al., 2022; Choudhary et al., 2014; Hyun et al., 2017). Small-molecule metabolites are closely associated with specific epigenetic modifications. For instance, various acyl-CoA donors can be catalyzed through a range of acylations by histone acetyltransferases (HATs) (Xie et al., 2024; Egger et al., 2024; He et al., 2023). Histone PTMs exert diverse effects on physiological and pathological processes. Typically, histone methylation and acetylation can impact histone–DNA binding affinity, alter chromatin accessibility, and regulate subsequent downstream gene transcriptions (Kuznetsova et al., 2020; Lawrence and Natoli, 2011; Sinha et al., 2023). In contrast, dysfunction of histone PTMs has been extensively studied in various diseases, including neurological disorders, inflammatory diseases, and cancers (Li et al., 2019; Netea et al., 2016; Noberini et al., 2018). Inhibition of H3K18/K27ac expression has been reported to significantly impact proliferation and metastasis of liver cancer cells (Cai et al., 2021). Histone deacetylase 3 (HDAC3) has been reported to be a crucial mediator of macrophage differentiation, activation, and polarization (Schultze, 2017; Van den Bossche et al., 2014). Comprehensive identification and quantitation of histone PTMs remain great challenges owing to several factors. First, histone PTMs exhibit a high degree of variability with more than 30 structurally distinct modifications, which are located on over 180 amino acid residues of histones (Millán-Zambrano et al., 2022; Zhao et al., 2024). Second, PTM crosstalk adds an additional layer of functional protein regulation and leads to expansion of the information content of a proteome. For instance, acetylation and methylation compete for the same site (H3K27) in embryonic stem cells (Huang et al., 2018; Ferrari et al., 2014). Third, histone PTMs are highly dynamic and reversible; histone PTMs with low abundances can further complicate identification and quantitation (Chan and Maze, 2020).

Mass spectrometry (MS)-based proteomics has advantages of high resolution, accuracy, and sensitivity, thereby providing the most accessible opportunity for global characterization of the histone PTM landscape (Cravatt et al., 2007). Among the MS-based quantitative strategies, stable isotope labeling by amino acids in cell culture (SILAC) is widely used in protein profiling and PTM analysis, where distinct isotopically encoded amino acids are introduced at the cellular level to reduce the experimental variability caused by sample preparation and improve quantitative accuracy (Kirchner and Selbach, 2012; Maile et al., 2015). Technological advancements from SILAC to super-SILAC have significantly expanded the quantitative capabilities of proteomics (Cuomo et al., 2011). The super-SILAC method utilizes a labeled mixture as an internal standard and is subsequently spiked into diverse samples for quantitative analysis (Noberini et al., 2016). This approach is beneficial for tissue samples and high-throughput sample sets. A standardized protocol for the super-SILAC method has been developed that utilizes treatment-naïve histone standard samples derived from various cell lines (Noberini and Bonaldi, 2017; Noberini et al., 2023). Tissue-specific histone propionylation and butyrylation were developed through derivatization methods in conjunction with super-SILAC, and compound stimulation has been suggested to increase the abundance of PTM peptides to facilitate further identification and quantitation (Vai et al., 2024).

In the present study, we optimized the super-SILAC method using multiple compound stimulations to increase the modification abundances of our histone standard samples. After assessing the identification depth, quantification reproducibility, and accuracy of our method at the synthetic peptide and whole-cell lysate levels, we applied the approach to M1 macrophages to elucidate the anti-inflammatory mechanism of LIQ. Quantitative transcriptomic results revealed the potential roles of histone PTMs in inflammatory processes and are expected to provide insights for further biological research on histone PTMs.

## Materials and methods

### Cell lines and reagents

The MCF-7, A549, MCF-10A, MDA-MB-231, THP1, and MDA-MB-468 cell lines used in this work were obtained from ATCC. The reagents used include DMEM for SILAC (cat. no. 88425, Thermo Fisher Scientific), dialyzed fetal bovine serum (FBS; cat. no. 30067334, GIBCO), ^13^C_6_-Lys (cat. no. 211204102, Silantes), ^13^C_6_-^15^N_4_-Arg (cat. no. 201604102, Silantes), protease inhibitor cocktail (cat. no. 6538282001, Roche), modified sequencing-grade trypsin (HLS TRY001N, HuaLishi Scientific), GSK-LSD1 dihydrochloride (GSK-LSD1·2HCl; S7574, Selleck), Jumonji histone demethylase inhibitor (JIB-04; S7281, Selleck), vorinostat or suberoylanilide hydroxamic acid (SAHA; HY-10221, MCE), nicotinamide (NAM; S1761, Beyotime), trichloroacetic acid (TCA; T0699, Sigma), Sep-Pak C18 cartridges (WAT023590, Waters), acetyl lysine antibody (ICP0380, Immunechem), methylated (ε-N) lysine antibody (ICP0501, Immunechem), horseradish peroxidase anti-histone H3 antibody (ab21054, Abcam), acetyl-histone H3 (Lys14) (D4B9) rabbit mAb (7627S, Cell Signaling), acetyl-histone H3 (Lys27) rabbit mAb (F0271, Selleck), anti-acetyl-histone H3 (Lys9) rabbit pAb (PTM-112, PTM Biolabs), anti-acetyl-histone H4 (Lys5) rabbit pAb (PTM-119, PTM Biolabs), acetyl-histone H4 (Lys16) (E2B8W) rabbit mAb (13534S, Cell Signaling), anti-histone H2B (acetyl K12) (Ab61228, Abcam), anti-histone H2A (acetyl K5) (Ab45152, Abcam), anti-monomethyl-histone H3 (Lys4) antibody (07-436, Millipore), trimethyl-histone H3 (Lys4) antibody (Cell Signaling, 9727S), dimethyl-histone H3 (Lys9) (D85B4) antibody (Cell Signaling, 4658S), trimethyl-histone H3 (Lys9) (D4W1U) antibody (Cell Signaling, 13969T), dimethyl-histone H3 (Lys27) rabbit mAb (F0329, Selleck), anti-trimethyl-histone H3 (Lys27) antibody (05-1951-S, Millipore), and dimethyl-histone H3 (Lys36) antibody (2901S, Cell Signaling).

### Peptides

The histone peptides were synthesized by Nanjing TGpeptide Biotechnology Co. Ltd. (Supplementary Table S1).

### Cell cultures

MCF-7, A549, MDA-MB-231, and MDA-MB-468 cells were cultured in DMEM containing 10% FBS (cat. no. 10091148, GIBCO) and 1% penicillin and streptomycin (CM-0525, Procell). The THP1 cells were cultured in RPMI 1640 medium containing 10% FBS and 1% penicillin and streptomycin solution. The MCF-10A cells were cultured with DMEM/F12 containing 5% horse serum (HS), 20 ng/mL of epidermal growth factor (EGF), 0.5 μg/mL of hydrocortisone, 10 μg/mL of insulin, 1% non-essential amino acids (NEAAs), as well as 1% penicillin and streptomycin. The THP1 cells were treated with 500 nM of phorbol myristate acetate (PMA) for 6 h, followed by treatment with 100 ng/mL of lipopolysaccharide (LPS) and 20 ng/mL of IFNγ for 12 h to activate the M1 type macrophages. Lastly, the M1-type cells were treated with LIQ for another 24 h. All cells were grown at 37°C supplied with 5% CO_2_.

### SILAC-based cell culture

For SILAC, the MCF-7 and A549 cells were labeled in DMEM with ^13^C_6_ lysine and ^13^C_6_^15^N_4_ arginine containing 2 mM of L-glutamine, 1% penicillin and streptomycin, and 10% dialyzed FBS (cat. no. 04-011-1A, Biological Industry) for 6–8 passages. The labeling efficiency was determined by liquid chromatography mass spectrometry (LC-MS/MS) analysis before histone extraction. When the labeling efficiency was above 95%, the cells were treated with 2.0 μM of GSK-LSD1·2HCl and 0.1 μM of JIB-04 for another 7 d; on the sixth day, 5 μM of SAHA and 10 mM of NAM were added to the medium for 24 h. After 7 d, the cells were harvested for histone extraction.

### Histone extraction

The harvested cells were lysed using an extraction buffer (10 mM of HEPES at pH 7.0, 10 mM of KCl, 1.5 mM of MgCl_2_, 0.34 M of sucrose, and 1× protease inhibitor cocktail) with 0.5% nonidet P-40 (NP-40) on ice for 30 min. After centrifugation, the pellets were washed with the extraction buffer without NP-40, resuspended in 0.2 M of H_2_SO_4_, and incubated overnight at 4°C. The supernatant was collected for TCA precipitation, and the precipitate was washed with cold acetone. The histone precipitate was dried at room temperature and dissolved in ddH_2_O.

### In-solution digestion

Histone was diluted in 100 mM of NH_4_HCO_3_. Then, sequence-grade trypsin was added at a trypsin-to-protein ratio of 1:50 and incubated overnight at 37°C. The sample was dried using SpeedVac. Lastly, the histone peptide sample was desalted using Sep-Pak C18 cartridges (Waters).

### In-gel digestion

Histone was separated in 12% gel and stained with Coomassie brilliant blue. Then, the histone bands were cut and gel particles were washed with 50% ethanol to remove the blue color. Next, the gels were washed with water twice for 20 min each, and the gel bands were cut into 1 mm^3^ pieces. The gels were dehydrated in 100% acetonitrile (ACN) until they shrank into white pieces and were then dried using a SpeedVac. Later, the gels were rehydrated with 10 ng/μL of trypsin solution in 50 mM of NH_4_HCO_3_ and incubated overnight at 37°C. We then washed the gels in 50 μL of 50% ACN for 15 min, 75% ACN/0.1% TFA for 15 min, and 100% ACN for another 5 min. The three extracts were finally combined and dried using the SpeedVac. The tryptic peptides were desalted using Sep-Pak C18 cartridges.

### LC-MS/MS analysis

The histone peptides were detected using the Orbitrap Ascend Tribrid™ mass spectrometer (Thermo Fisher Scientific) coupled with the Vanquish Neo high-performance liquid chromatography (HPLC) system (Thermo Fisher Scientific). The peptides were dissolved in 0.1% formic acid in water (v/v), centrifuged at 21,130*g* for 10 min, and the supernatant was injected into a self-made capillary liquid chromatography column filled with C18 resin. The flow phase A contained 0.1% formic acid in water (v/v), while the flow phase B included 0.1% formic acid, 90% ACN, and 10% water. The gradient elution steps were as follows: 0–7 min, 0%–5% B; 7–36 min, 5%–12% B; 36–101 min, 12%–30% B; 101–111 min, 30%–45% B; 111–120 min, 45%–80% B. Then, the eluted peptides were analyzed using the Ascend mass spectrometer, which was used for full MS scans from 300 to 1500 m/z with a resolution of 120,000. In the data-dependent mode, the precursor ions with charge states 1–5 were selected for higher energy collisional dissociation (HCD) fragmentation at 30% collision energy, and the secondary fragment ions were detected using Orbitrap with the dynamic exclusion time set to 10 s.

### Analysis of histone PTMs

The histone PTMs were analyzed using Mascot software (version 2.3.01) and the UniProt human histone database. Trypsin/P was selected as the enzyme with five missed cleavages. The fixed modifications include label:13C(6)(K) and label:13C(6)15N(4) (R), while the variable modifications include oxidation (M), label:13C(6)(K) + methyl, label:13C(6)(K) + dimethyl, label:13C(6)(K) + trimethyl, label:13C(6)(K) + acetyl, label:13C(6)(K) + propionyl, label:13C(6)(K) + butyryl, label:13C(6)(K) + crotonyl, label:13C(6)(K) + malonyl, label:13C(6)(K) + succinyl, label:13C(6)(K) + GlyGly (ubiquitination), label:13C(6)(K) + lactyl, label:13C(6)(K) + hydroxyisobutyryl, label:13C(6)15N(4) (R) + dimethyl, label:13C(6)15N(4) (R) + trimethyl, and phospho (S/T). The peptide tolerance was 10 ppm and MS/MS tolerance was 0.02 Da. The analyses were conducted for five representative histone variants, including P0C0S8 (H2A1), P0C0S5 (H2A.Z), P62807 (H2B1C), P84243 (H3.3), and P62805 (H4).

### Quantitative analysis of histone PTMs

A comprehensive wildcard database search was conducted using Byonic software (Protein Metrics Inc.) with the following parameter configurations. The precursor mass tolerance was set at 6 ppm, and the fragment ion mass tolerance was defined as 0.5 Da. Trypsin/P was designated as the digestion enzyme, and up to five missed cleavages were permitted. Data analyses were performed against the human histone sequence database derived from the UniProt database (version 2023-06-08, comprising 86 sequences). The fixed modifications include label:13C(6)(K) and label:13C(6)15N(4) (R); the variable modifications were label:13C(6)(K) + methyl, label:13C(6)(K) + dimethyl, label:13C(6)(K) + trimethyl, label:13C(6)(K) + acetyl, label:13C(6)(K) + propionyl, label:13C(6)(K) + butyryl, label:13C(6)(K) + crotonyl, label:13C(6)(K) + succinyl, label:13C(6)(K) + glutaryl, label:13C(6)(K) + GlyGly (ubiquitination), label:13C(6)(K) + lactyl, label:13C(6)(K) + hydroxyisobutyryl, label:13C(6)15N(4)(R) + methyl, label:13C(6)15N(4)(R) + dimethyl, and phospho (S/T). A Byonic score threshold of 250 was applied (Bern et al., 2009; Pirro et al., 2021; Riley et al., 2019), and only the peptide spectra with sequence lengths exceeding five residues were retained for analyses. For peptides identified with only heavy labels, we complemented their light-labeled data through *in silico* methods using Byonic. The valence states identified across unique peptides were integrated to ensure comprehensive representation. All quantitative analyses were conducted for six representative histone variants, including P0C0S8 (H2A1), P0C0S5 (H2A.Z), P62807 (H2B1C), P84243 (H3.3), P68431 (H3.1), and P62805 (H4). All spectra and ion-current integration regions were manually checked to ensure reliability prior to quantification.

### RT-qPCR

The total RNA was extracted from the cells using the RNA extraction kit (Abclonal RK30120), and cDNA was obtained using the reverse transcription kit (Abclonal RK20429). Quantitative PCR (qPCR) was then performed using the SYBR Green Fast qPCR Mix (Abclonal RK21203) on the Bio-Rad system (Supplementary Table S2).

### Western blotting (WB)

Appropriate proteins or histones were lysed with 2× loading buffer (100 mM of Tris-Cl, 4% SDS, 0.2% bromophenol blue, 20% glycerol, and 200 mM of DTT), and the solution was boiled at 99°C for denaturation. Then, approximately 2.0 μg of the protein or histone was separated using a polyacrylamide gel before transferring to a nitrocellulose membrane. The membrane was then blocked and immunoblotted with appropriate antibodies, before washing and incubating with peroxidase-conjugated secondary antibodies. Lastly, the membranes were washed and imaged by chemiluminescence (Clinx, chemiscope 6000).

### RNA isolation and library preparation

RNA was extracted from M1 macrophages treated with 30 μM of LIQ (three control and three LIQ groups) using TRIzol reagent according to manufacturer protocols (Invitrogen, CA, United States). Then, RNA purity and quantification were achieved using the NanoDrop 2000 spectrophotometer (Thermo Fisher Scientific). The RNA integrity, library construction, transcriptome sequencing, and analysis were conducted by OE Biotech Co., Ltd. (Shanghai, China).

### Statistical analysis

Statistical analyses were performed using GraphPad Prism software. All data were presented as mean ± standard error of the mean (SEM). Unpaired two-tailed Student’s t-test with equal variance was performed. The statistical details of each experiment are shown in the figure legends. The significance is indicated with symbols as follows: **p < 0.01 or ^##^ *p* < 0.01, *p < 0.05, and not significant: *p* > 0.05.

## Results

### Optimization of the super-SILAC strategy

**FIGURE 1:**
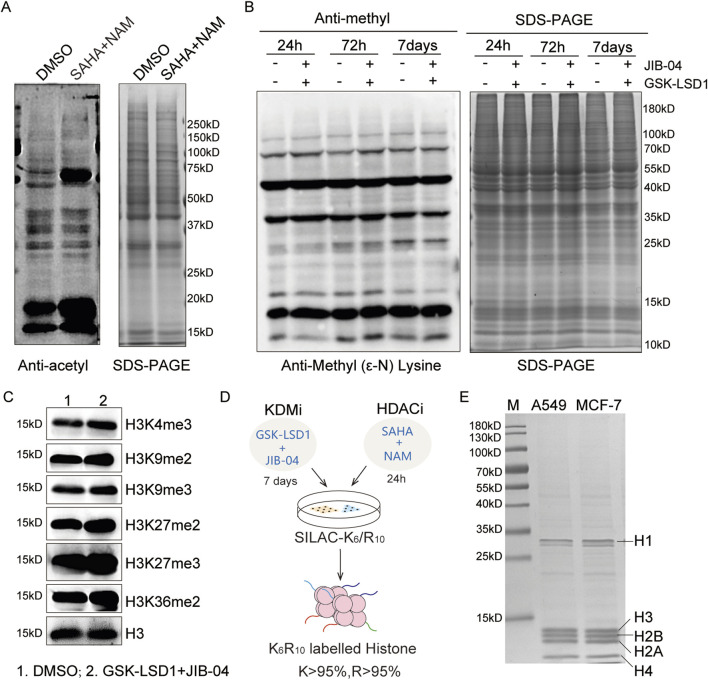
Optimization of the super-SILAC strategy: **(A)** immunoblot analysis of the global acetylation levels after treatments with SAHA (5.0 μM) and NAM (10 mM) for 24 h; **(B, C)** immunoblot analyses of the global and site-specific methylation levels following treatments with GSK-LSD1·2HCl (2.0 μM) and JIB-04 (0.1 μM); **(D)** workflow for the optimized super-SILAC strategy; **(E)** SDS-PAGE analysis of the purity of heavy histones extracted from A549 and MCF-7 cells.

HDACs are divided into the Rpd3/Hda1 and sirtuin families, which function through either zinc-dependent or NAD^+^-dependent mechanisms (Seto and Yoshida, 2014; Yang and Seto, 2008). Vorinostat (SAHA) is the first marketed inhibitor of the Rpd3/Hda1 family of HDACs, and NAM is an effective biochemical inhibitor of sirtuins (Anderson et al., 2003; Gallo et al., 2004; Bitterman et al., 2002). The synergistic effects of SAHA and NAM can significantly increase histone acetylation levels (Figure 1A). Similarly, lysine-specific demethylase (LSD) and Jumonji C (JMJC) are two evolutionarily conserved families of histone demethylases that have different reaction mechanisms. The LSD family is demethylated into monomethylated and dimethylated substrates by the flavin adenine dinucleotide (FAD)-dependent amine oxidation reactions, and the JMJC family id demethylated into mono-, di-, and tri-methylated residues through Fe (II) and α-ketoglutarate (Bitterman et al., 2002).

We combined the two compounds GSK-LSD1·2HCl and JIB-04 to increase the histone methylation levels. Owing to the slower dynamics of methylation compared to acetylation, no significant changes were detected over a short period of time (Zee et al., 2010). We then explored the time-dependent changes in methylation levels, which showed that the histone methylation sites were upregulated after the 7-day treatment, especially at the H3K4me3, H3K9me3, H3K27me2, H3K27me3, and H3K36me2 modification sites (Figures 1B, C). Therefore, we employed GSK-LSD1·2HCl and JIB-04 to treat the cells for 7 d, where SAHA and NAM were added over the last 24 h to prepare the SILAC histone standard samples (Figure 1D). SDS-PAGE results indicate that the histone standard samples have high purity (Figure 1E). Based on increased levels of acetylation and methylation modifications, we obtained heavy-labeled histone mixture standards with labeling efficiencies of K > 95% and R > 95%.

### Histone standard samples improve identification coverage of histone PTMs

**FIGURE 2:**
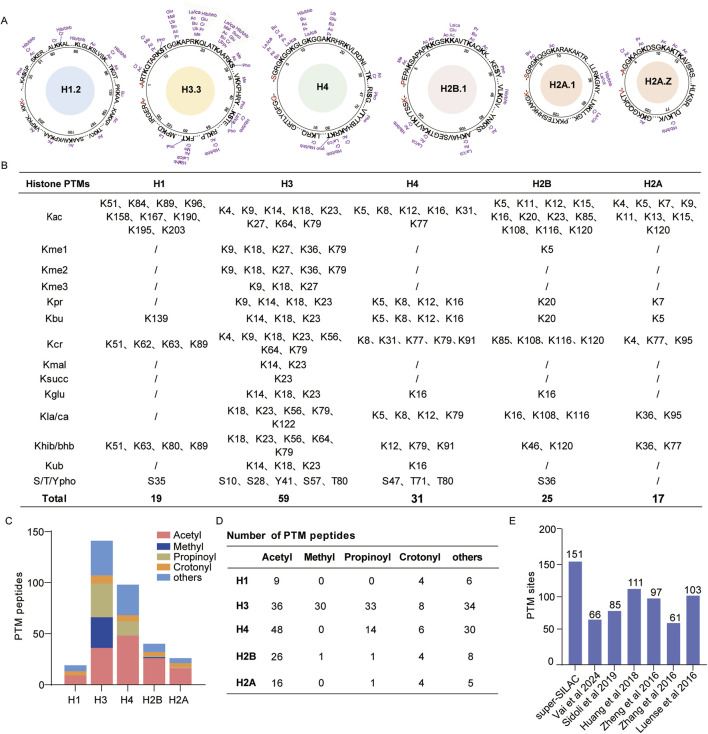
Deep identification of histone post-translational modifications (PTMs) by super-SILAC; **(A)** map of histone PTM sites identified by in-gel digestion; **(B)** list of identified histone PTM sites; **(C, D)** global distribution of histone PTM peptides; **(E)** number of histone PTM sites identified in this study and comparison with those of other histone PTM profiling studies. Abbreviations: lysine acetylation (Kac), monomethylation (Kme1), dimethylation (Kme2), trimethylation (Kme3), propionylation (Kpr), butyrylation (Kbu), crotonylation (Kcr), malonylation (Kmal), succinylation (Ksucc), ubiquitination (Kub), lactylation/carboxyethylation (Kla/Kca), 2-hydroxyisobutyrylation/β-hydroxybutyrylation (Khib/Kbhb), arginine monomethylation (Rme1), arginine dimethylation (Rme2), and serine/threonine/tyrosine phosphorylation (S/T/Ypho).

To evaluate qualitative coverage, we utilized the in-gel digestion approach to collect H1, H2A, H2B, H3, and H4 of our histone standard samples. Following LC-MS/MS analysis, the data were analyzed against the UniProt human histone sequence database using Mascot software, which helped identify 12 modification types and 151 modification sites (Figure 2A; Supplementary Table S1). Among these, we found 59 histone PTM sites located on the N-terminal of H3, nine different modification sites on H3K18, and 10 different modification sites on H3K23. In addition, we identified a total of 123 acylation sites containing 42 acetylation sites and 81 other novel acylation sites (Figure 2B). The identified PTM peptides comprised 135 acetylated, 49 propionylated, and 26 crotonylated peptides (Figures 2C, D). Consequently, the diversity of the above modification sites and peptides can assist with subsequent qualitative and quantitative analyses.

We compared our results with the findings of other proteomics-based histone PTM profiling studies. Vai et al. (2024) identified 66 histone PTM sites in clinical breast cancer tissue with the optimized histone in-gel derivatization procedure. Sidoli et al. (2019) identified 85 histone PTM sites using the SILAC method in the murine T lymphoblast cell line EL4. Huang et al. (2018) identified 111 histone PTM sites in six cancer cell lines using in-gel digestion. Zheng et al. (2016) identified 97 histone PTM sites through in-gel digestion in mouse embryonic stem cells and neural progenitor cells. Luense et al. (2016) obtained 103 histone PTM sites in human germ cells using propionylation labeling and trypsin digestion. Compared with these results, our optimized super-SILAC strategy shows deeper peptide coverage and more histone modification sites (Figure 2E). The proposed compound stimulation strategy enhances the abundances of various histone PTMs, especially those with low abundances, which allows an in-depth profiling dataset of the histone PTMs.

### Optimized super-SILAC approach shows good quantitative performances at the peptide and cellular lysate levels

**FIGURE 3:**
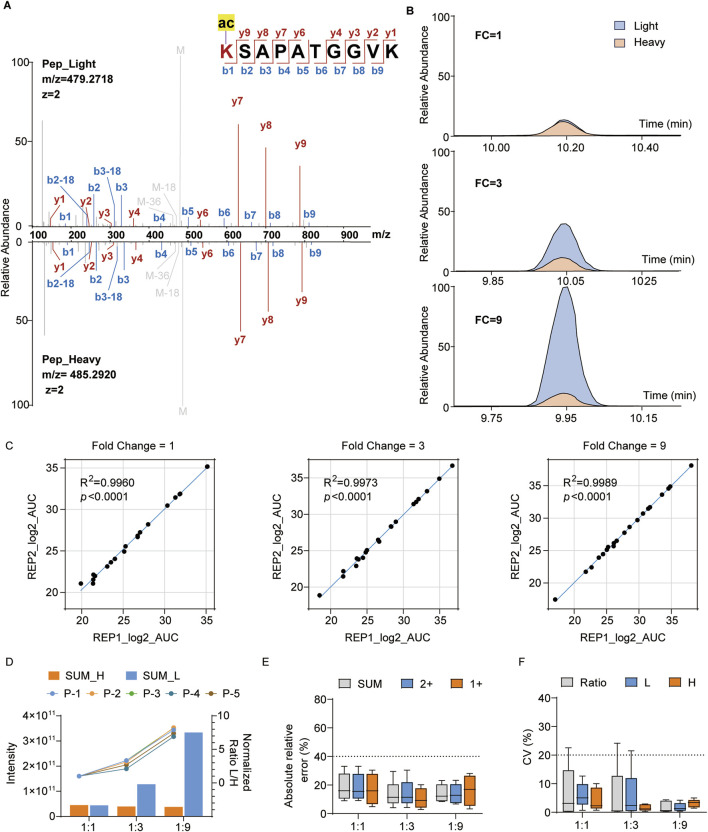
Quantification of the synthesized histone modification peptides with heavy histone standards: **(A)** representative MS/MS spectrum of the light-labeled synthesized peptides and heavy-labeled histone peptides with the sequence KSAPATGGVK (H3K27ac); **(B)** relative abundances of the representative peptides in **(A)** at different mixing ratios (blue: light-labeled peptide; yellow: heavy-labeled peptide); **(C)** correlation analysis between two replicates at different mixing ratios (REP1: replicate 1, REP2: replicate 2); **(D)** quantification intensities of the peptides and normalized ratios across different groups; **(E, F)** absolute relative errors and coefficient of variation (CV) values for different groups.

We initially synthesized five histone-modified peptides, namely, H3K18ac, H3K23ac, H3K27ac, H3K23pr, and H3K18bu/K23ac, to evaluate the quantitative reproducibility and accuracies of our histone standard samples. The purity of these peptides was verified by LC-MS/MS, and the results show that the heavy-labeled histone standard peptide and light-labeled synthesized peptide exhibit high-quality and nearly consistent retention times of the MS/MS spectra (Figures 3A, B). Based on the purities of the five synthesized peptides, we combined them into a mixture with specific proportions to ensure approximately equal quantitative areas under the curve (AUCs) in the extracted ion chromatograms (XICs); then, the histone standard peptides were mixed with above synthesized peptide mixture at ratios of 1:1, 1:3, and 1:9 (Figure 3B). The quantitative results show high correlation (R > 0.99) between the replications in different groups, demonstrating good reproducibility (Figure 3C; Supplementary Table S2). Furthermore, we evaluated the normalized ratio and absolute relative error of each peptide, whose results show consistent quantitative trends with the mixing ratios (Figure 3D) and less than 30% error compared to the theoretical values (Figure 3E). The coefficient of variation (CV) values of all groups were below 20%, indicating relatively low variability (Figure 3F). Thus, our histone standard samples demonstrate effective reproducibility and high accuracies for quantitative analysis at the peptide level.

**FIGURE 4:**
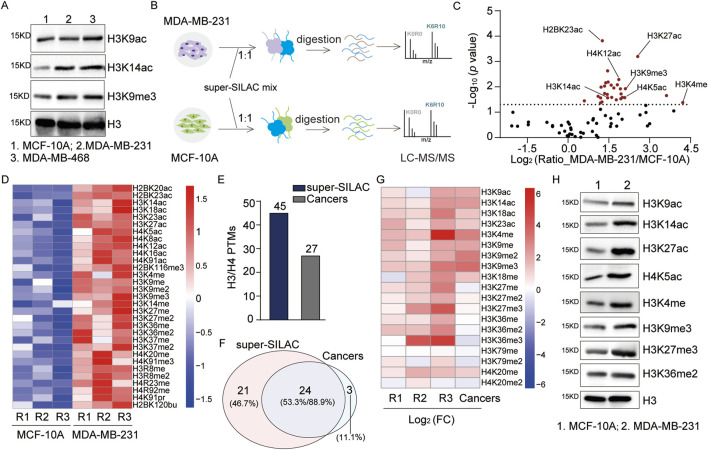
Systematic analysis of the histone PTMs in breast cancer cells: **(A)** immunoblot analyses of the levels of H3K9ac, H3K14ac, and H3K9me3 in breast cancer and non-tumorigenic breast epithelial cells; **(B)** workflow for the systematic analysis of histone PTM sites in breast cancer cells; **(C)** scatter plot analysis of the differential histone PTM sites (red dots *p* < 0.05); **(D)** heatmap analysis of the quantitative sites with *p*-values <0.05 in MCF-10A and MDA-MB-231 groups based on three replicates (R1: replicate 1, R2: replicate 2, R3: replicate 3); **(E, F)** comparative analysis of the numbers and overlaps of H3/H4 PTM sites identified in this study with those from a previous study by Noberini et al. (2018); **(G)** comparative heatmap analysis of the quantitative H3/H4 PTM sites identified by Noberini et al. (2018); **(H)** immunoblot analysis of specific histone PTM sites in breast cancer and non-tumorigenic breast epithelial cells.

It is widely acknowledged that histone PTMs play crucial regulatory roles in cancer. Extensively studied histone marks, such as H3K9me3, H3K9ac, and H3K14ac, that are associated with the activation of gene expression (Liu et al., 2023) exhibit consistent increasing trends in Luminal A-like, Luminal B-like, and triple-negative breast cancer, suggesting that they could be used as potential biomarkers (Noberini et al., 2019). Similarly, our WB results show consistent upregulation of these sites in triple-negative breast cancer cells (MDA-MB-468 and MDA-MB-231) relative to non-tumorigenic breast epithelial cells (MCF-10A) (Figure 4A). Some authors have systematically analyzed histone PTMs in breast cancer cell lines through the super-SILAC method (Cuomo et al., 2011; Noberini et al., 2018; Noberini et al., 2019); however, their works have only focused on histone PTMs of H3 and H4. Therefore, we aimed to systematically assess the differences between cancer cells and ordinary epithelial cells to further evaluate our approach in a complex sample system.

By applying the optimized super-SILAC method, we extracted histones from MCF-10A and MDA-MB-231 cell lines as well as performed quantitative analyses of the histone PTMs (Figure 4B). Compared to non-tumorigenic breast epithelial cells, a total of 82 histone PTM sites were quantified (Figure 4C; Supplementary Table S3A), which contained 31 significantly upregulated sites (*p* < 0.05) (Figure 4D). In addition to previously reported upregulated sites, such as H3K4me, H3K9ac, and H4K14ac, we quantified two uncharacterized sites, namely, H4K91pr and H2BK120bu, which were also significantly upregulated (*p* < 0.05) (Figure 4D). Focusing on H3 and H4, we quantitatively analyzed 45 sites that encompass the majority of sites identified by Noberini et al. (2018) (Figures 4E, F; Supplementary Table S3B). The comparison of overlapping identified sites showed consistent quantitative results for most sites (Figure 4G). For further validation, we assessed the variation trends of the PTM sites via WB, which were consistent with the above quantitative results (Figure 4H). In conclusion, our optimized super-SILAC method for quantitative analysis demonstrates high reliability at both the peptide and cellular lysate levels. These results highlight the potential of our histone standard samples in elucidating disease pathology and drug mechanisms.

### Epigenetic regulation of histone PTMs by LIQ in M1 macrophages

**FIGURE 5:**
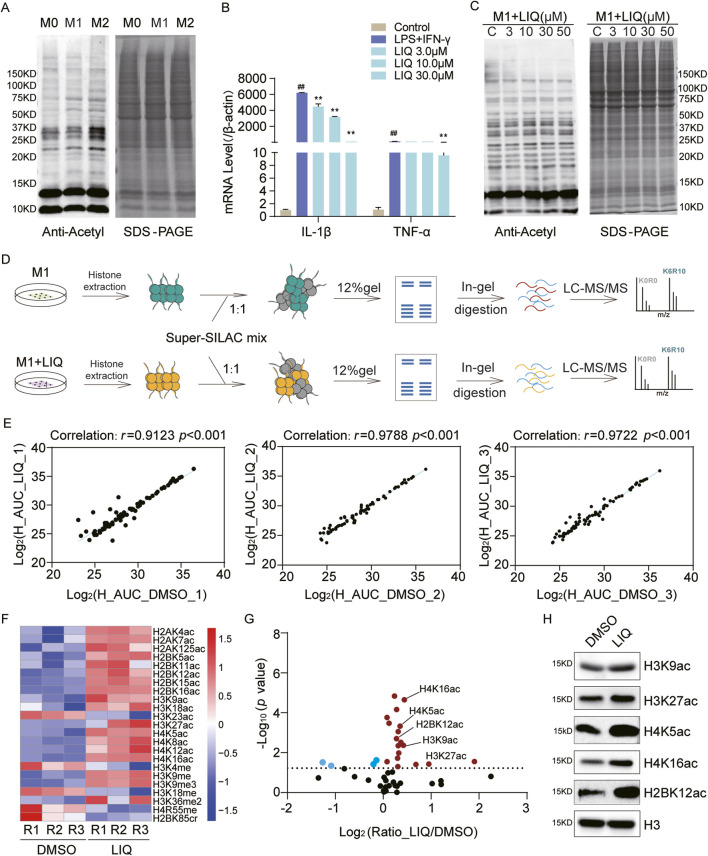
Systematic analysis of histone PTMs regulated by liquiritigenin (LIQ) in macrophages; **(A)** immunoblot analysis of the global acetylation levels in M0, M1, and M2 phenotype macrophages. **(B)** RT-qPCR analysis of the IL-1β and TNFα mRNA expressions following LIQ treatment from three replicates; mean ± standard error of the mean (SEM) (n = 3), ^##^ *p* < 0.01 compared with the control group, **p < 0.01 compared with the lipopolysaccharide + IFN-γ group; **(C)** immunoblot analysis of the global acetylation levels in M1 macrophages treated with LIQ; **(D)** workflow for the identification of LIQ-mediated histone PTM sites in M1 macrophages; **(E)** correlation analysis of the heavy peptides between dimethyl sulfoxide (DMSO) and LIQ groups based on three replicates; **(F)** heatmap analysis of the quantitative histone PTM sites with *p* < 0.05 across the replicates; **(G)** scatter plot analysis of the differential histone PTM sites, where red dots represent up-regulated sites and blue dots represent down-regulated sites (*p* < 0.05); **(H)** immunoblot analysis of the specific histone PTM sites after LIQ treatment.

Histone PTMs, especially acetylation, enable regulation of the transcriptional repression of macrophage inflammatory responses (Shi et al., 2024). The THP1 cells, which are commonly used to model macrophage differentiation, were induced into the M0, M1, and M2 phenotypes; their acetylation levels were assessed by pan-acetylation antibodies, and the results revealed a significant downregulation of acetylation in M1 macrophages and marked upregulation in M2 macrophages (Figure 5A).

LIQ is a dihydroflavonone monomer found in licorice and has been widely demonstrated to exert natural anti-inflammatory effects (Babu et al., 2023; Tu et al., 2019; Yu et al., 2015). To explore the epigenetic regulatory mechanisms of LIQ, we treated M1 macrophages with varying concentrations of LIQ (3, 10, 30, and 50 μM), with dimethyl sulfoxide (DMSO) as the control. The qPCR results showed that LIQ treatment significantly inhibited the expressions of inflammatory factors (IL-1β and TNF-α) in M1 macrophages (Figure 5B). Furthermore, statistical analysis of the inflammatory factors revealed significant downregulation of IL-1β, IL-R1, NOS2, and TNF (Supplementary Figure S1C), indicating potential anti-inflammatory effects of LIQ. WB revealed that LIQ treatment upregulated histone acetylation levels in a concentration-dependent manner (Figure 5C), suggesting that LIQ may modulate inflammatory responses through the regulation of histone PTMs.

We next applied super-SILAC to identify LIQ-mediated histone PTMs in M1 macrophages (Figure 5D; Supplementary Table S4). Comparisons of the DMSO and LIQ groups across three biological replicates revealed a high degree of correlation (r > 0.91) (Figure 5E). A total of 49 histone PTM sites were quantified, which were mapped to 15 PTM types. We found that most of the acetylation sites and several methylation sites were upregulated in the LIQ-treated groups (*p* < 0.05) (Figure 5F). Scatter plot analysis revealed significant upregulation of these sites across all three replicates (*p* < 0.05), such as H3K9ac, H3K27ac, H4K5ac, H4K16ac, and H2BK12ac (Figure 5G). WB analysis confirmed consistency with the MS analysis results (Figure 5H). The above findings highlight that LIQ can potentially regulate epigenetic modification alterations to participate in anti-inflammatory mechanisms.

### LIQ may regulate the peroxisome proliferator-activated receptor (PPAR) signaling pathway to exert anti-inflammatory function

**FIGURE 6:**
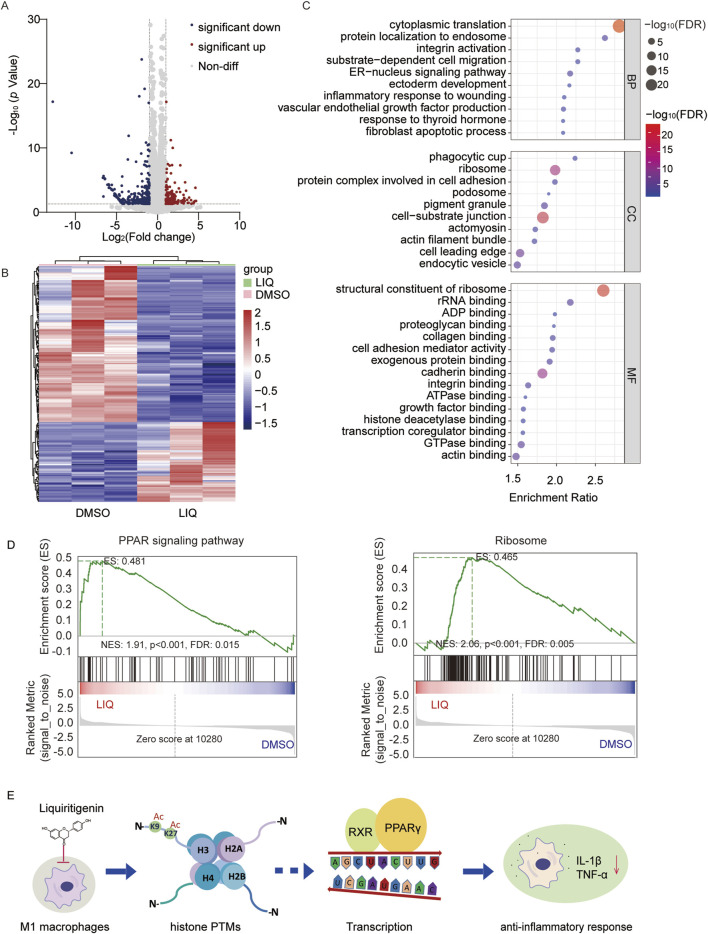
LIQ regulates gene expressions through the peroxisome proliferator-activated receptor (PPAR) signaling pathway in anti-inflammatory processes. **(A)** Volcano plot of the genes identified in M1 macrophages following LIQ treatment; the red and blue dots represent significantly upregulated and downregulated genes, respectively, while the gray dots represent non-differential genes; representative differentially expressed genes (-log_10_ *p* = 0–30, *p* < 0.05) are displayed, and all significant data are shown in Supplementary Figure S2. **(B)** Cluster analysis of the differentially expressed genes (fold change >2.0 and *p* < 0.01) in the DMSO and LIQ groups. **(C)** Gene Ontology (GO) analysis of the related genes with *p* < 0.05, showing top-10 biological processes (BPs) and cellular components (CCs) as well as top-15 molecular functions (MFs). **(D)** Gene set enrichment analysis (GSEA) through comparisons with the KEGG database for the PPAR signaling pathway and ribosome enrichment in the LIQ groups (FDR <0.05). **(E)** Proposed mechanisms underlying the anti-inflammatory effects of LIQ.

LIQ treatment resulted in changes to the histone PTM landscape in macrophages, suggesting that it could affect the expressions of certain genes. Volcano plot analysis and principal component analysis (PCA) of the altered genes revealed significant differences between the LIQ-treated and control groups (Figure 6A; Supplementary Figure S1A; Supplementary Table S5A). A total of 473 differentially expressed genes (DEGs) were identified based on a significance threshold of *p* < 0.05 and fold change >2.0 (Supplementary Table S5B). Among these, 157 genes were significantly upregulated, whereas 316 genes were significantly downregulated (Figure 6B; Supplementary Figure S1B). Gene ontology (GO) molecular function analysis revealed that LIQ affected the structural constituents of the ribosomes, rRNA binding, and histone deacetylase binding (false detection rate (FDR) <0.05). In addition, these DEGs were involved in biological processes such as translation, protein localization, inflammatory responses, and miRNA metabolism. The GO cellular components analysis showed that these genes were primarily located in the ribosomes and protein complexes (FDR <0.05) (Figure 6C).

Gene set enrichment analysis (GSEA) was used to identify the significantly enriched pathways, such as PPAR signaling and ribosome pathways (FDR <0.05) (Figure 6D). The PPAR signaling pathway is reportedly involved in anti-inflammatory responses. Moreover, activation of the PPARs may influence acetylase activity, and changes to the acetylation levels could in turn modulate PPAR signaling (Zeng et al., 2022). Considering the upregulation of histone acetylation by LIQ, we also examined the genes related to deacetylases. The results showed significant downregulation of deacetylases, such as HDAC4, HDAC5, HDAC8, HDAC10, SIRT2, SIRT3, and SIRT7, indicating that their genes may influence epigenetic regulation by mediating alterations in deacetylase upon LIQ treatment (Supplementary Figure S1D). In summary, transcriptome analysis revealed that LIQ may exert anti-inflammatory effects by modulating the histone PTMs and regulating gene expressions through the PPAR signaling pathway (Figure 6E).

## Discussion

Given their planar ring structures, dihydroflavonones exhibit a broad spectrum of biological activities, encompassing anti-inflammatory and antitumor effects. LIQ is a dihydroflavonone monomer compound derived from natural products. However, the relationships between its anti-inflammatory effects and epigenetic regulatory mechanisms remain unclear (Hu et al., 2017; Chagas et al., 2022). In the present study, we optimized the super-SILAC method through compound stimulations and found that LIQ could exert anti-inflammatory activity by regulating the histone PTMs and gene expressions through the PPAR signaling pathway in M1 macrophages. The optimized method provides a powerful tool for the epigenetic study of natural products and offers insights into the epigenetic mechanisms of LIQ.

Compared with previous research, we improved the abundances of the PTM peptides through compound stimulations. Based on quantitative analyses at the peptide and cellular lysate levels, we demonstrated high accuracy and reproducibility of our histone standard samples. Moreover, the optimized method was applicable to both cell and tissue sample types. Notably, the quantitative accuracy of high-abundance modifications was relatively higher than that of low-abundance modifications at the synthesized peptide level. At the cellular lysate level, the shared identified sites between the findings of Noberini et al. (2018) and our results showed consistent trends for most sites, and discrepancies were observed only in a few sites, such as H4K20me3 and H4K16ac, which could be attributed to variations in the cell line cultures or MS detection method. Notably, the widespread upregulation highlights aberrant epigenetic regulation in breast cancer cells, potentially driving the expressions of oncogenes. The underlying mechanisms of this phenomenon merit further exploration.

Histone acetylation and methylation are associated with transcriptional activation and chromatin structure alterations (Shahbazian and Grunstein, 2007). We found that LIQ upregulated the acetylation and methylation sites, such as H3K9ac, H3K27ac, H4K5ac, H4K16ac, and H2BK12ac, in M1 macrophages. Thus, we hypothesized that LIQ exerted anti-inflammatory function through epigenetic regulation. Through transcriptome analysis, we found that LIQ significantly affected the PPAR signaling pathway, which is involved in lipid metabolism and inflammatory responses (Daynes and Jones, 2002; Ahmadian et al., 2013; Han et al., 2017; Maréchal et al., 2018). PPAR activation affects deacetylase activity, enhancing H3 acetylation and promoting macrophage polarization (Liu et al., 2020; Wang et al., 2022); alteration of the acetylation level thus regulates PPAR signaling, which is cooperatively involved in the regulation of gene expression (Zeng et al., 2022). Furthermore, H3K27ac directly affects the expression of PPARγ by enhancing its transcriptional activity (Duan et al., 2022). The increase in H3K9ac/14ac positively regulates the transcriptional activity of PPARγ (Małodobra-Mazur et al., 2024), and PPARγ activation is an indispensable factor in maintaining the acetylation levels of H3K9 and H3K27 (Yuan et al., 2021). All of the above evidence further confirms that the anti-inflammatory mechanisms of LIQ are closely related to histone epigenetic regulation and the PPAR signaling pathway. In addition, we found that the *XIST* and *CD200* genes were silenced by LIQ, and several transcription factors like the RXRG, KLF4, FOXP3, and ERG were significantly regulated (*p* < 0.05, fold change >2.0) (Supplementary Table S5). Thus, the detailed mechanisms by which LIQ silences *XIST*, *CD200*, and their transcription factors need further investigation. In summary, the findings of this study advance our understanding of the anti-inflammatory mechanisms of dihydroflavonones in macrophages and provide a strategy for future research on the epigenetic mechanisms of natural products.

## Data Availability

The original contributions presented in this study are included in the article/Supplementary Material, and any further inquiries may be directed to the corresponding authors.
