# Supplementary material for: Quantitation of global histone post-translational modifications reveal anti-inflammatory epigenetic mechanisms of liquiritigenin based on the optimized super-SILAC strategy
Source: Front Cell Dev Biol. 2025 Mar 27;13:1566567. doi: 10.3389/fcell.2025.1566567 (PMC11982745; doi:10.3389/fcell.2025.1566567)
Supplement: Supplementary file 1 [file DataSheet1.zip › Supplementary Materials.docx]

***Supplementary Material***

**Supplementary Figures**


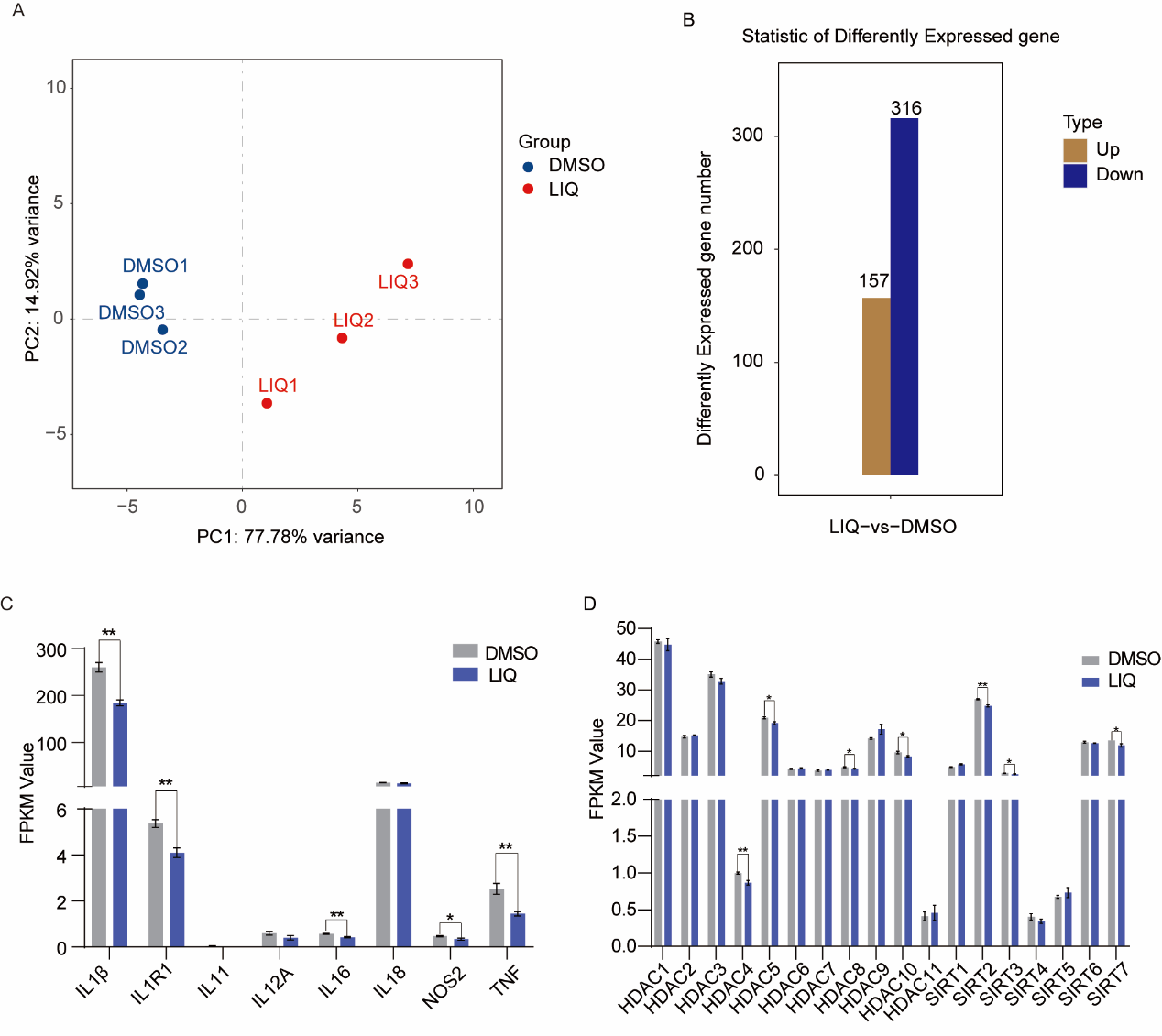


**Supplementary Figure 1.** LIQ regulates gene expression through PPAR signaling pathway in anti-inflammatory process. **(A)** Principal Component Analysis (PCA) of LIQ and DMSO groups, three replicates. **(B)** The number of differential genes in LIQ vs DMSO (fold change > 2.0, and *p* < 0.05). **(C)** The histogram analysis of related inflammatory factors. ***p* < 0.01, **p* < 0.05. **(D)** The histogram analysis of related deacetylase genes. ***p* < 0.01, **p* < 0.05.


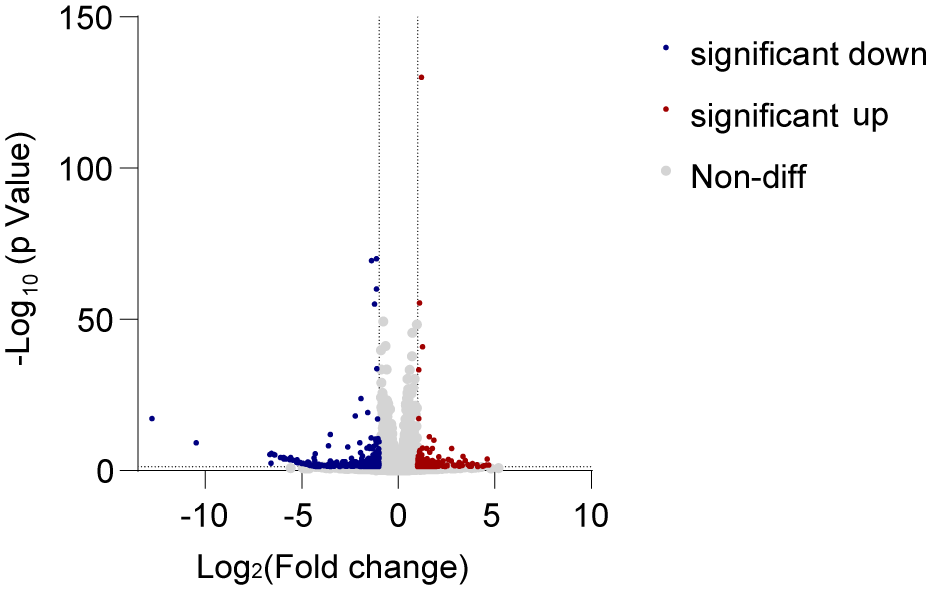


**Supplementary Figure 2.** Volcano plot analysis of the identified genes in M1 macrophages following LIQ treatment. Display all of the significant genes (-log_10_ *p*, *p* < 0.05).

**Supplementary Tables**

| **Sites** | **Peptides** | **Purity** |
| --- | --- | --- |
| H3K18ac | K(ac)QLATK | 97.24% |
| H3K23ac | QLATK(ac)AAR | 97.32% |
| H3K27ac | K(ac)SAPATGGVK | 97.13% |
| H3K23pr | QLATK(pr)AAR | 95.23% |
| H3K18bu/K23ac | K(bu)QLATK(ac)AAR | 97.58% |

**Supplementary Table 1.** Purity of synthetic peptides used for verification of quantitative accuracy of standards.

**Supplementary Table 2.** Primer sequences used for QPCR.

| Primers | | Sequence (5’-3’) |  |
| --- | --- | --- | --- |
| β-actin | Forward | CATGTACGTTGCTATCCAGGC |  |
|  | Reverse | CTCCTTAATGTCACGCACGAT |  |
| TNF-α | Forward | CCTCTCTCTAATCAGCCCTCTG |  |
|  | Reverse | GAGGACCTGGGAGTAGATGAG |  |
| Il-1β | Forward | TTCGACACATGGGATAACGAGG |  |
|  | Reverse | TTTTTGCTGTGAGTCCCGGAG |  |
